# Development of Nano–Micro Fused LSPR Chip for In Situ Single-Cell Secretion Analysis

**DOI:** 10.3390/mi14071404

**Published:** 2023-07-11

**Authors:** Yuhei Terada, Ain Obara, Jonathan Campos Briones, Xi Luo, Wilfred Villariza Espulgar, Masato Saito, Hyota Takamatsu, Eiichi Tamiya

**Affiliations:** 1 Environmental Management Research Institute (EMRI), Department of Energy and Environment, National Institute of Advanced Industrial Science and Technology (AIST), Tsukuba 305-8569, Ibaraki, Japan; 2 Advanced Photonics and Biosensing Open Innovation Laboratory, AIST-Osaka University, Suita 565-0871, Osaka, Japan; 3 Department of Applied Physics, Graduate School of Engineering, Osaka University, Suita 565-0871, Osaka, Japan; 4 Life and Medical Photonics Division, Institute for Open and Transdisciplinary Research Initiatives, Osaka University, Suita 565-0871, Osaka, Japan; 5 Department of Respiratory Medicine and Clinical Immunology, Graduate School of Medicine, Osaka University, Suita 565-0871, Osaka, Japan; 6 Laboratory of Autoimmune Diseases, Department of Clinical Research Center for Autoimmune Diseases, NHO Osaka Minami Medical Center, Kawachinagano 586-8521, Osaka, Japan; 7 SANKEN, Osaka University, Ibaraki 567-0047, Osaka, Japan

**Keywords:** nano/micro fabrication technique, single-cell-level immunoassay, cytokine detection, nano–micro fused structure, localized surface plasmon resonance (LSPR) technique

## Abstract

Single-cell analysis has become increasingly important in uncovering cell heterogeneity, which has great implications in medicine and biology for a deep understanding of cell characteristics. Owing to its significance, it is vital to create novel devices that can reveal special or unique cells. In this work, we developed a single-cell secretion detection chip consisting of microwells that can trap single cells. Each well is surrounded by Au nanopillars capable of localized surface plasmon resonance (LSPR) measurement. Using microfabrication and nanofabrication techniques, Au nanopillar and microwell structures were fabricated on a COP film. The Au nanopillar was modified with IL-6 antibodies for the direct detection of single-cell secreted IL-6 via LSPR absorbance peak shift. Specific IL-6 detection was successfully demonstrated using a null and IL-6 oversecreting Jurkat cell. A high single-cell trapping efficiency of over 80% was also achieved. Overall, the development of this single-cell secretion detection chip with a simple LSPR measurement setup represents a significant development in the field of cell biology and immunology, providing researchers with a powerful tool for studying individual cells and their secreted cytokines, and is useful for point-of-care testing (POCT) diagnostics.

## 1. Introduction

Single-cell secretion analysis has become increasingly important because of its ability to detect and characterize secreted molecules from individual cells that could not have been achieved by bulk or average analysis. This enables the identification of cells that are secreting different types or levels of molecules and thus provides a more comprehensive view of cellular activity and heterogeneity. This has many potential applications such as disease biomarker identification, immune response characterization, and cellular communication mechanisms study. In cancer research, for example, single-cell analysis can be used to identify cells that exhibit elevated levels of cytokine secretion, potentially contributing to tumor growth.

Cytokines are cell-secreted immunological proteins with relatively small molecular weights (6–70 kDa) [1]. It is an attractive biomarker because of its involvement in cell signaling, cell differentiation, and the human immune system [2,3,4]. For example, interleukin-6 (IL-6) is a cytokine belonging to the interleukin group of signaling molecules that plays a critical role in the immune system, particularly in regulating the growth and activity of immune cells. IL-6 is a key mediator of the inflammatory response, the body’s natural defense mechanism against infection and injury [5,6,7,8]. Elevated levels of IL-6 have been linked to the pathogenesis of many inflammatory diseases [9].

One of the common and widely used biosensing methods for highly sensitive cytokine detection is the enzyme-linked immunosorbent assay (ELISA) [10,11,12,13]. While ELISA allows for the highly sensitive detection of cytokines, it necessitates the labeling of proteins and secondary antibodies, leading to a complex workflow with multiple experimental steps and posing challenges for high-throughput analyses. To overcome such problems, enzyme-linked immunospot (ELISPOT) assay can be utilized [14,15,16,17]. ELISPOT offers a high-throughput, quantitative means of measuring single-cell cytokine secretion, exhibiting higher detection sensitivity. However, one notable drawback of the ELISPOT assay in single-cell analysis is its limited sensitivity and precision when detecting low-frequency antigen-specific T cells. In addition, the spot size of the ELISPOT assay can vary depending on the number of cytokines secreted by the captured cell, leading to variability in the measurement [18]. Another issue is the problem of human counting errors, which can impact the accuracy of the detection results [19,20].

Microfluidics technology, on the other hand, is a powerful tool for single-cell secretion analysis. Several microfluidic techniques have been developed for cell-based assays such as droplet microfluidics, hydrodynamic trapping, PDMS, and hydrogel-based nanowells, etc. For instance, Chokkalingam et al. made use of a droplet-based approach to detect cytokine secretion from single cells that are encapsulated in monodisperse agarose droplets together with functionalized cytokine-capture beads [21]. However, this process requires additional steps, including droplet collection, gelling, emulsion breaking, and flow cytometry sorting and analysis, which introduce complexity to the detection system. Microchamber oil encapsulation has also been reported for single-cell compartmentalization and cytokine secretion detection and monitoring [22]. Being a closed system, it is difficult to retrieve cells of interest for additional analysis (i.e., RNA seq.). George and Wang developed a splittable microchip that integrates a high-density antibody array for single-cell cytokine detection [23]. However, the utilization of antibodies or antibody barcodes in a sandwich ELISA assay can be expensive, as it necessitates specialized materials during fabrication and carries the risk of cross-reactivity and nonspecific binding. Meanwhile, Choi et al. fabricated monolithic hydrogel nanowells within a microwell plate using laser patterning to confine cytokine secretion from single cells [24]. Here, the utilization of expensive lasers for micropatterning, along with the requirement of capture beads and labeled antibodies, increases the cost of chip production.

The plasmonic biosensor represents an attractive candidate for cytokine detection, offering label-free, real-time, and rapid measurement of biomolecular interactions such as antigen–antibody reactions, carbohydrate–protein interactions, and nucleic acid hybridization [25,26,27,28,29]. Particularly, the localized surface plasmon resonance (LSPR) technique simplifies the detection of these biological interactions by assessing optical changes, such as shifts in scattering intensity spectrum and absorbance spectrum, resulting from refractive index (RI) changes induced by biomolecular interactions near metal nanostructures [26,29,30,31,32]. Moreover, LSPR biosensors necessitate a relatively straightforward optical setup, making them valuable for point-of-care testing (POCT) diagnostics [33,34]. Recently, the feasibility of two-dimensional imaging measurement at the single-cell level has been reported [35,36]. However, further enhancements are necessary to improve its throughput. To apply this technique in single-cell-level analysis, the integration of LSPR nanostructures with microstructures for cell trapping is required [32,37,38]. Incorporating microfluidics allows for the control of cell migration and cell-to-cell interactions by modifying trap structures and the spacing between microwells [39,40,41,42,43,44]. Currently, several studies have demonstrated the integration of LSPR format with microfluidics for diagnostics or cell analysis, but the nanostructures for facilitating LSPR and the microstructures for cell trapping had to be prepared separately [32,45,46,47]. Consequently, these two critical structures had to be combined afterward, which presents a challenge in device preparation.

Our group previously reported the development of an LSPR biosensor chip with a nanopillar structure. The chip was prepared by thermal nanoimprinting of a porous anodic alumina mold onto a cyclo-olefin polymer (COP) film, followed by Au sputtering [48]. This biosensor chip demonstrated the capability to detect both antigen–antibody reactions and carbohydrate–protein reactions in dry and wet conditions [48,49,50]. In this research, we have further developed an LSPR biosensing microfluidic chip that incorporates microwells for single-cell capture and nanopillar structures on the same chip surface surrounding the wells to capture secretions in situ. Through microfabrication and nanofabrication techniques, Au nanopillar and microwell structures were fabricated on a COP film. The Au nanopillar was functionalized with IL-6 antibodies to enable direct detection of IL-6 secreted by single cells via LSPR absorbance peak shift. Furthermore, a high single-cell trapping efficiency of over 80% was achieved. This proof-of-concept device has the potential to specifically detect single-cell secreted cytokines and significantly impact various fields, including medicine and biology, by providing a deeper understanding of cell characteristics, immune response, and drug development.

## 2. Materials and Methods

### 2.1. Fabrication of Nanopillar Structure onto the COP Plate

The nanopillar chip was fabricated for the detection of biomolecular interactions using LSPR. An Au layer was deposited onto the nanopillar using a sputtering method. A detailed procedure for nanopillar fabrication was previously reported in our studies [45,46]. Meanwhile, a COP plate (Zeonex5000, Zeon Corporation, Tokyo, Japan) with nanopillar structure was prepared using nano-imprint lithography (NIL). First, a porous alumina mold was created using a two-step anodizing method to replicate nanopillar structures onto the COP plate. A polished alumina substrate was initially cleaned by sonication with acetone, followed by MilliQ water, and then dried using an N_2_ air gun. The substrate was immersed in an etching solution (80% *w*/*v* phosphoric acid and 5% *w*/*v* chromic acid) at 80 °C for 30 min. Subsequently, it was rinsed with MilliQ water and placed in a 1 M oxalic acid solution. The first anodizing process was conducted at 80 V for 1 h. After rinsing with MilliQ water and drying with N2 gas, the substrate was once again incubated in the etching solution for 30 min at 80 °C, followed by sonication in MilliQ water and drying with N2 gas. The second anodizing process was performed at 80 V for 13 s. Subsequently, the substrate was sonicated with MilliQ water and dried using N2 gas. Finally, it was immersed in a 1.6% phosphoric acid aqueous solution for 12 min at 40 °C, followed by sonication in MilliQ water and drying with N2 gas.

The prepared alumina mold was utilized in NIL using an X-300H nanoimprinting system (SCIVAX Corp., Kawasaki, Japan). To maintain balanced applied pressure, the alumina mold and COP plate were sandwiched between silicon wafers. Prior to the NIL step, the silicon wafers and alumina mold were treated with a 1% fluorine coating agent diluted in perfluoro-*i*-hexane. The temperature of the nanoimprinting stage was set to 60 °C, and a pressure of 150 N was applied for 1 min. The chamber was then evacuated to −90 kPa. Subsequently, the temperature of the lower stage was raised to 80 °C, and a pressure of 3.56 MPa was applied for 10 min. Afterward, the stages were allowed to cool down to 40 °C, and the applied pressure was reduced to 150 N. Finally, the vacuum chamber was brought back to atmospheric pressure, and the COP plate was removed. The plate was subsequently cut into appropriate chip sizes and used in subsequent experiments.

### 2.2. Refractive Index (RI) Sensitivity Measurement of the Nanopillar Chip

For the measurement of absorbance spectra, the nanopillar chip was placed into a flow cell (FLAB50-UV-02 flow cell, GLSciences Inc., Tokyo, Japan), and light from a tungsten halogen light source (LS-1, GLSciences Inc., Tokyo, Japan) was vertically irradiated onto the chip using an optical fiber probe bundle (P400-2-UV/VIS, GLSciences Inc., Tokyo, Japan). The absorbance spectra were obtained from the transmitted light, which was measured by a conventional spectrometer (USB4000, wavelength range: 200–1100 nm, GLSciences Inc., Tokyo, Japan).

The absorbance spectrum peak of the light irradiated vertically onto the Au-sputtered nanopillar chip (Au thickness: 20 nm) was measured in various refractive index (RI) environments: air (*n* = 1.00), water (*n* = 1.33), 1 M glucose aqueous solution (*n* = 1.35), and ethylene glycol (*n* = 1.43). The peak wavelength obtained in each environment was plotted against the corresponding RI values. Linear approximation was then applied to the obtained plots, and the slope of the line represented the bulk refractive index of the nanopillar chip.

### 2.3. Fabrication of Nano–Micro Fused Structure

A microwell mold, which would be used for imprinting microwells onto the nanopillar chip, was prepared using deep reactive ion etching (DRIE). A 2 mL positive photoresist (AZ4330 diluted three times in MilliQ water) was dispensed onto a 4-inch silicon wafer and spin-coated at 3000 rpm for 30 s. The wafer, shielded from light, was baked for 1 min at 65 °C and then at 90 °C. Next, the wafer was exposed to a UV light source with an intensity of 1000 J/m^2^ using a maskless lithography machine (D-Light DL-1000, PMT, Fukuoka, Japan) and allowed to cool down for 10 min. It was then immersed in a developing solution for 40 s and rinsed with MilliQ water. Subsequently, chromium (Cr) was sputtered onto the wafer under the following conditions: 8 mL/min argon flow rate, 50 W radio frequency output, 5 min of presputtering time, and 10 min of sputtering time. The resist was then removed by sonication in acetone for 10 min. The DRIE process was performed on the wafer using the Bosch process with 800 cycles.

Next, the protective layer for the nanopillar structure was prepared. A 2 mL SU-8 3005 photoresist was poured onto the wafer and spin-coated at 2700 rpm for 30 s. The formed SU-8 layer was exposed to UV light through a film mask for 5 s using an MA-10 mask aligner (Mikasa, Tokyo). The wafer was then baked for 3 min at 65 °C and 10 min at 95° C. Subsequently, the wafer was immersed in a developing solution for 5 min and rinsed with isopropanol.

Finally, the microwell structure was transcribed onto the nanopillar chip using the NIL system. The temperature of the stage was set to 55 °C, and the nanopillar chip was placed on the microwell mold wafer. Sandwiched between silicon wafers, the chip and the mold were treated with a 1% fluorine coating agent diluted in perfluoro-*i*-hexane. The imprinting step was performed under a pressure of 20 N for 5 min to parallelize the nanopillar chip and the microwell mold, then pressed with a force of 600 N for 5 min.

### 2.4. Cell Trapping Measurement Using the Nano–Micro Fused Chip

First, a Jurkat cell suspension was prepared. Jurkat cells were incubated in an RPMI media with 10% FBS for 3 days. Media exchange was performed by pelleting the cells via centrifugation at 900 rpm for 5 min. The supernatant was then aspirated, and an HBSS solution with 0.1% BSA and a fluorescent dye (CSFE, ThermoFisher, ex/em = 492/517 nm) was added. The cells were stained by incubating the suspension for 20 min. The suspension was centrifuged again to replace the media, and HBSS with 1% BSA was added. The cell concentration was determined using a C-Chip hemocytometer.

Prior to the test, a 20 nm thick layer of Au was sputtered onto the prepared nano–micro fused chip. The chip was then covered with a silicon sheet and a cover glass to create a flow channel. The Au surface was blocked by injecting a 30 µL solution of 1 µM BSA in PBS into the flow channel and incubating it for 45 min. The remaining BSA was washed off by flowing PBS into the channel. Next, a 100 µL cell suspension (3.2 × 10^6^ cells/mL) was injected. After incubating for 1 min, the suspension was drawn out, and a 200 µL solution of HBSS with 0.1% BSA was injected twice. The trapped cell was determined via fluorescence imaging, and the percentage of single-cell well trapping was calculated by dividing the total number of trapped cells number by the number of microwells, multiplied by 100. To determine the optimal well size for single-cell trapping based on the target cell sample, we varied the well diameter. Each observable area of the chip consisted of 100 microwells with well diameters of 9, 10, 11, and 12 µm. We used four areas, each containing 100 microwells, for every measurement.

### 2.5. Cytokine Secretion Detection of the Trapped Cell by LSPR Measurement

For the immobilization of antibody, succinimide–amine coupling was used. The Au-sputtered nano–micro fused chip was rinsed with EtOH and incubated in a 2 mM solution of 10-carboxydcanethiol in EtOH solution for 30 min. The chip was rinsed with EtOH and water, followed by drying with N_2_ gas. Subsequently, a solution containing 100 mg/mL of NHS and 100 mg/mL of WSC was introduced onto the chip and incubated for 30 min. The chip was then rinsed with water and dried with N_2_ gas. The NHS-activated chip was quickly covered with a silicon sheet and cover glass, after which a 30 µL quantity of 1 µM anti-IL-6 solution was injected and incubated for 45 min. The chip was washed with PBS to remove the unbound antibodies. Subsequently, the chip was blocked by injecting a 500 µL solution of 1 M monoethanolamine hydrochloride (pH 8.5) and incubating for 1 h. Finally, the chip was washed with PBS.

A 100 µL amount of 3.0 × 10^6^ cell/mL Jurkat cell suspension was injected into the chip and incubated for 1 min. It was then washed twice with a 200 µL PBS solution. For identification of detection specificity, two types of cells (null Jurkat cells and IL-6 overexpressed Jurkat cells) were used. Spectral images were obtained every 5 min for 55 min using a hyperspectral imaging system (SIS-1n-es, EBA JAPAN Co., Ltd., Tokyo, Japan) equipped with a microscope (IX70, Olympus, Japan) and an XY-electrical stage (BIOS-225T-0L-IQ, Andor, Tokyo, Japan). The absorbance peak wavelengths of each pixel were calculated from the spectral images. Here, the absorbance was calculated by comparing the transmitted light intensity through the LSPR-active sample with a reference measurement. The measurement area was designated as the region of interest (ROI), specifically configured around the microwells. The time course of the absorbance peak position at each ROI was obtained by averaging the spectrum within the ROI. Due to noise interference, accurate peak calculation for this absorption spectrum was challenging. To address this, a 9-term polynomial fitting was applied.

## 3. Results and Discussion

### 3.1. Morphology of the Nano–Micro Fused Chip

**Figure 1:**
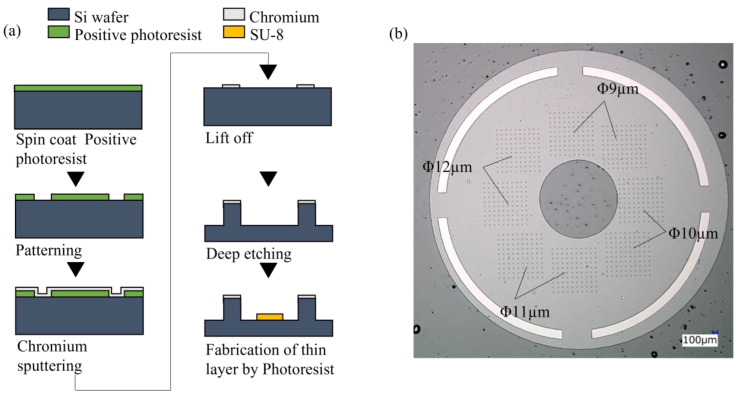
Microwell mold fabrication: (**a**) schematic illustration of the microwell mold preparation; (**b**) microscopic image of the microwell mold with varying cylindrical structures (⌀: 9, 10, 11, 12 µm) clustered in an area and distributed on the mold surface as indicated. Each area has 100 (10 × 10) microstructures.

**Figure 2:**
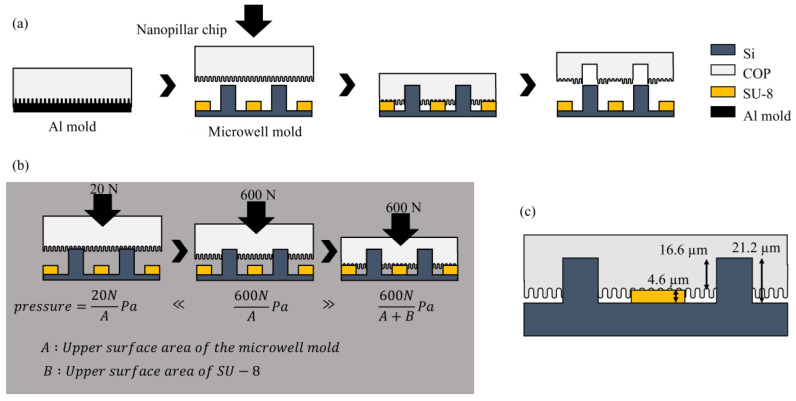
Schematic illustration of the nano–micro fused chip fabrication: (**a**) transcription of the microwell structure onto the nanopillar chip using the NIL system; (**b**) detailed illustration of the imprint step. A 20 N force was applied to parallelize the nanopillar chip and microwell mold. A 60 N force was then applied to transcribe the microwell structure. (**c**) dimension specification of the mold microstructures and resulting microwell.

The schematic illustrations of the overall procedure for the nano–micro fused chip fabrication are described in Figure 1 and Figure 2. Periodic cylindrical microstructures were successfully created on the Si wafer mold to be used for printing microwell onto the COP nanopillar chip (Figure 1). During NIL, the Si wafer mold was pressed on the COP nanopillar chip. Said mold had an SU-8 layer to prevent the collapse of the nanopillar structures. As illustrated in Figure 2, the SU-8 layer had a height of 4.6 µm, and the resulting microwells on the COP chip had a depth of 16.6 µm and a 10 µm average diameter, which was large enough for trapping single Jurkat cells (⌀: 8–12 µm) [42].

**Figure 3:**
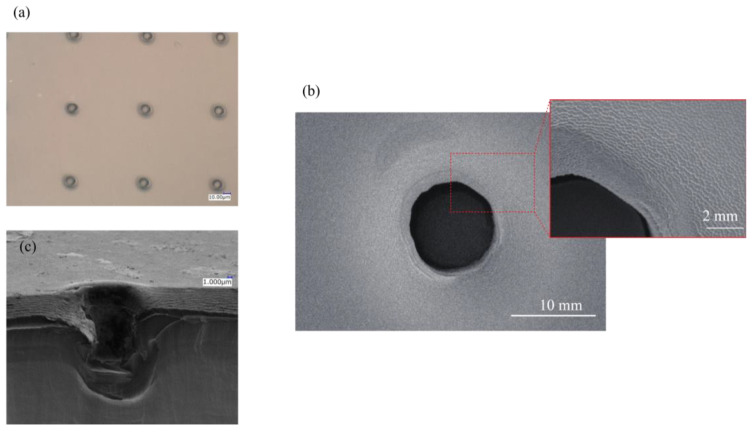
Transcribed microwell on the nano–micro fused chip: (**a**) optical microscope image of microwell array; (**b**) SEM image of a single microwell, inset: magnified view of the surface showing the nanostructure surface; (**c**) cross-sectional SEM image of a microwell.

Figure 3 shows the scanning electron microscope (SEM) image of the nano–micro fused chip and its microwell array (Figure 3a). A microwell hole with about 10 µm in diameter surrounded by nanopillars can be clearly observed in Figure 3b. As shown in the enlarged view of the SEM image, a slight deformation was observed on the surface with nanopillar structures around the well area, approximately 2 µm from the microwell edge, resulting from the imprint process (formation of slope). This depression on the nanopillar surface may lead to a change in LSPR measurement; hence, the absorbance spectra outside of this inclined area was set as the measurement area for the cytokine adsorption detection.

### 3.2. Refractive Index Sensitivity of the Nanopillar Chip

**Figure 4:**
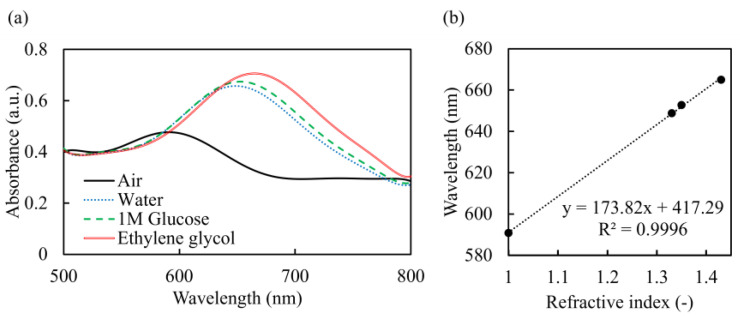
Refractive index sensitivity of the nanopillar chip: (**a**) absorbance spectra; (**b**) absorbance peak top of the Au-capped nanopillar chip in different media.

The absorbance spectra of Au-sputtered nanopillar chip in different RI environments, and the absorbance peak wavelength at each environment, are summarized in Figure 4. A red shift in the absorbance spectrum was observed as the RI at the Au nanopillar surface increased from 1.00 (air) to 1.43 (ethylene glycol). It was clearly shown that the prepared Au nanopillar chip exerted the LSPR phenomenon. The absorbance peak wavelength at each environment showed a linear relationship with a slope of 173.8 nm/RIU (refractive index unit). This value that was obtained was defined as the RI sensitivity, which was found to be relatively high compared to previously reported Au nanopillar chip [48]. This high RI sensitivity is estimated to be sufficient for detecting antigen–antibody reactions [48].

### 3.3. Cell Trapping Efficiency of the Nano–Micro Fused Chip

**Figure 5:**
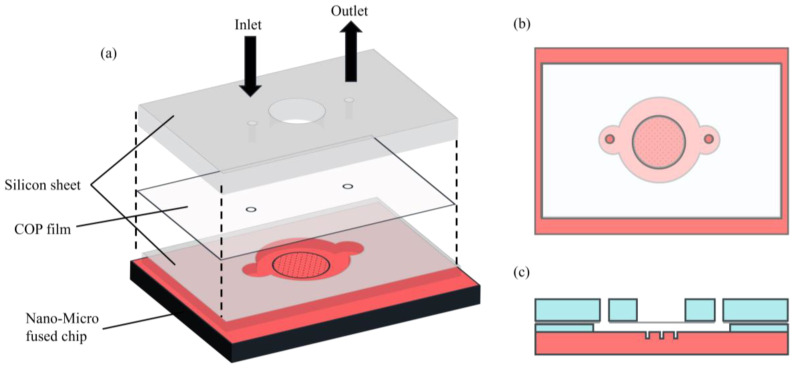
Schematic illustration of the cell trapping setup of the nano–micro fused chip: (**a**) by-layer illustration of the chip showing the setup’s composition; (**b**) top view showing the microwell area, inlet, and outlet; (**c**) side view showing the cross section and the flow channels of the chip.

The single-cell trapping efficiency of the nano–micro fused chip with different microwell diameter was evaluated using a setup described in Figure 5 and Figure S1. The cell suspension was injected from the inlet and pumped out of the outlet using a micropipette. Adherence of cells to the upper surface of the chip with nanopillars was prevented through BSA blocking, ensuring that the injected cells were individually trapped in the microwells.

**Figure 6:**
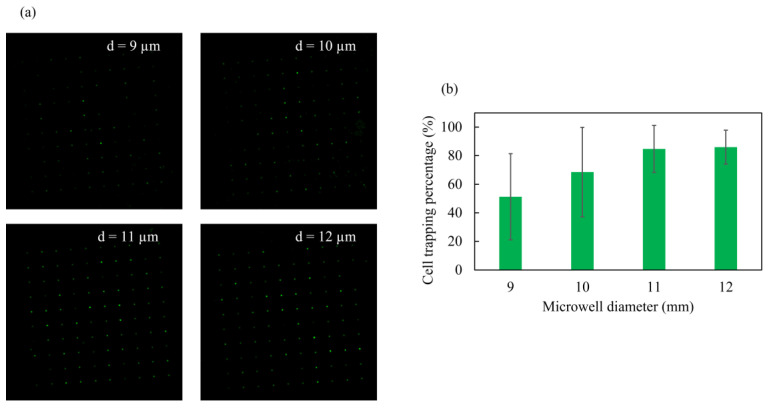
Single-cell trapping efficiency of the nano–micro fused chip: (**a**) fluorescent images of the trapped stained cells in different microwell diameter; (**b**) percentage of single-cell trapping by well size (N = 4).

The percentage of single-cell trapping was obtained through cell counting from the fluorescent images shown in Figure 6a. The percentage of single-cell well trapping was calculated by dividing the total number of trapped cells by the number of microwells, multiplied by 100. The single-cell trapping percentage of the chip with well diameter of 9, 10, 11, and 12 µm was computed to have a mean (N = 4) of 51, 69, 85, and 86%, respectively. It was observed that the cell trapping ratio increased as the microwell diameter increased. The chip with a microwell diameter of 12 µm showed the highest single-cell trapping efficiency with a smaller standard deviation, std.dev. = 11.92 (Figure 6b). Thus, it was determined that the 12 µm well size was suitable for single-cell trapping of the target Jurkat cell (⌀: 8–12 µm). A diameter larger than 12 µm was not explored, as it might lead to the trapping of multiple cells in a microwell.

### 3.4. Single-Cell Cytokine Secretion Detection by LSPR Measurement

**Figure 7:**
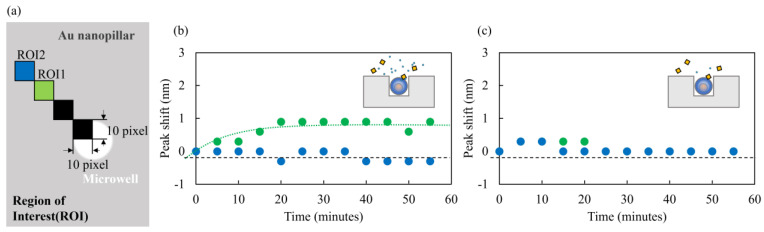
Time course of the absorbance peak shift: (**a**) ROI measurement location around the microwell indicated as ROI1 (green) and ROI2 (blue); (**b**) time dependency of the LSPR peak shift by adsorption of IL-6-overexpressing Jurkat cell; (**c**) normal or null Jurkat cell secretion.

The absorbance peak shifts around the microwells with IL-6 overexpressing Jurkat cell and null Jurkat cell were evaluated. The ROI was configured as depicted in Figure 7a, and the measurement of absorbance peak shift arising from the adsorption of IL-6 onto the antibody-immobilized Au-capped nanopillar surface was conducted. The absorbance peak shift was measured for ROI1 and ROI2, excluding the deformed slope of nanopillars observed at the microwell’s edge, as previously discussed and shown in Figure 3b.

The time course of the absorbance peak shift is presented in Figure 7b for IL-6 overexpressing cells and Figure 7c for null cells. In the case of IL-6 overexpressing Jurkat cells, a red shift of the absorbance peak was observed in ROI1, indicating the adsorption of the biomolecule onto the Au-capped nanopillar surface. However, no absorbance peak shift was observed in ROI2. We speculate that the local concentration of the secretion was higher near the microwell (ROI1), while the number of molecules reaching ROI2 was likely below the detection limit. In the case of null Jurkat cells, no absorbance peak shift was observed in either ROI. Based on these results, it can be inferred that the adsorption of IL-6 onto the Au-capped nanopillar surface was successfully detected, and the binding was specific, as evidenced by the observed LSPR peak shift only in IL-6 overexpressing Jurkat cells.

The results highlight the capability of the developed device in effectively trapping single cells and specifically detecting in situ IL-6 secretion using LSPR. To further enhance the device, efforts are underway to improve the fabrication of the microwells, aiming to eliminate the slight deformation at the well edge and the formation of sloped nanopillar surfaces. This improvement will enable the configuration of the ROI closer to the well’s edge, thereby increasing sensitivity. As a proof-of-concept device, it has demonstrated the potential for specific detection of biomolecules secreted by individual cells in a simple LSPR measurement setup. Consequently, it provides researchers and clinicians with a powerful tool for studying individual cells and offers potential applications in point-of-care testing (POCT) diagnostics.

## 4. Conclusions

In this study, a nano–micro fused chip was developed using NIL and DRIE techniques for single-cell trapping and in situ detection of cell-secreted cytokines. The trapping efficiency for a 12 µm diameter microwell was achieved at 86%. The bulk refractive index (RI) sensitivity of the LSPR-based Au nanopillar was determined to be 178.3 nm/RIU, indicating its sensitivity for detecting single-cell cytokine secretion. The adsorption of secreted cytokines resulted in an observable LSPR peak shift at the surrounding Au nanopillar surface of the microwell. Notably, the LSPR response was only observed in the microwell containing IL-6 oversecreting Jurkat cells, confirming the specific detection of cytokines.

The results have demonstrated the utility of the developed device in detecting single-cell secretion. The simplicity of the LSPR measurement setup eliminates the complexities associated with other detection systems, as it requires only a limited number of steps for cytokine detection. Moreover, as an open-type device, specific cells of interest can be easily retrieved for further testing or downstream analysis. The use of NIL on COP film also offers a cost-effective approach for chip fabrication and even enables mass production. In conclusion, this proof-of-concept device has significant potential in point-of-care testing and related clinical diagnostics, making a substantial impact in the fields of medicine, biology, and research.

## Data Availability

The data presented in this study are available on request from the corresponding author.

## Supplementary Materials

The following supporting information can be downloaded at: https://www.mdpi.com/article/10.3390/mi14071404/s1, Figure S1: Schematic illustration of the of nano-micro fused chip set-up for cell trapping.

## References

[1] Stenken J.A., Poschenrieder A.J. (2015). Bioanalytical chemistry of cytokines—A review. Anal. Chim. Acta.

[2] O’Shea J.J., Murray P.J. (2008). Cytokine Signaling Modules in Inflammatory Responses. Immunity.

[3] Tisnocik J.R., Korth M.J., Simmons C.P., Farrar J., Martin T.R., Katze M.G. (2012). Into the Eye of the Cytokine Storm. Microbiol. Mol. Biol. Rev..

[4] Liu G., Qi M., Hutchinson M.R., Yang G., Goldys E.W. (2016). Recent advances in cytokine detection by immunosensing. Biosens. Bioelectron..

[5] Fishman D., Faulds G., Jeffery R., Mohamed-Ali V., Yudkin J.S., Humphries S., Woo P. (1998). The effect of novel polymorphisms in the interleukin-6 (IL-6) gene on IL-6 transcription and plasma IL-6 levels, and an association with systemic-onset juvenile chronic arthritis. J. Clin. Investig..

[6] Heinrich P.C., Behrmann I., Haan S., Hermans H.M., Müller-Newen G., Schaper F. (2003). Principles of interleukin (IL)-6-type cytokine signalling and its regulation. Biochem. J..

[7] Kishimoto T. (2005). Interleukin-6: From Basic Science to Medicine-40 Years in Immunology. Annu. Rev. Immunol..

[8] Sheller J., Chalaris A., Schmidt-Arras D., Rose-John S. (2011). The pro- and anti-inflammatory properties of the cytokine interleukin-6. Biochim. Et Biophys. Acta.

[9] Hirano T. (2021). IL-6 in inflammation, autoimmunity and cancer. Int. Immunol..

[10] Helle M., Boeije L., de Groot E., de Vos A., Aarden L. (1991). Detection of IL-6 in biological fluids: Synovial fluids and sera. J. Immunol. Methods.

[11] Steffen M.J., Ebersole J.L. (1996). Sequential ELISA for Cytokine Levels in Limited Volumes of Biological Fluids. BioTechniques.

[12] Wiese R., Belosludtsev Y., Powdrill T., Thompson P., Hogan M. (2001). Simultaneous Multianalyte ELISA Performed on a Microarray Platform. Clin. Chem..

[13] Prakash N., Stumbles P., Mansfield C. (2013). Initial Validation of Cytokine Measurement by ELISA in Canine Feces. Open J. Vet. Med..

[14] Okamoto Y., Murakami H., Nishida M. (1997). Detection of Interleukin 6-Producing Cells among Various Organs in Normal Mice with an Improved Enzyme-Linked Imuunospot (ELISPOT) Assay. Endocrine J..

[15] Ekerfelt C., Ernerudh J., Jenmalm M.C. (2002). Detection of spontaneous and antigen-induced human interleukin-4 responses in vitro: Comparison of ELISPOT, a novel ELISA and real-time PCR. J. Immunol. Methods.

[16] Kalyuzhny A.E. (2005). Chemistry and biology of the ELISPOT assay. Handbook of ELISPOT.

[17] Cox J.H., Ferrari G., Janetzki S. (2006). Measurement of cytokine release at the single cell level using the ELISPOT assay. Methods.

[18] Ma J., Peng Z., Ma L., Diao L., Shao X., Zhao Z., Liu L., Zhang L., Huang C., Liu M. (2022). A Multiple-Target Simultaneous Detection Method for Immunosorbent Assay and Immunospot Assay. Anal. Chem..

[19] Fujihashi K., McGhee J.R., Beagley K.W., McPherson D.T., McPherson S.A., Huang C.-M., Kiyono H. (1993). Cytokine-specific ELISPOT assay−Single cell analysis of IL-2, IL-4 and IL-6 producing cells. J. Immunol. Methods.

[20] Okamoto Y., Gotoh Y., Tokui H., Mizuno A., Kobayashi Y., Nishida M. (2000). Characterization of the Cytokine Network at a Single Cell Level in Mice with Collagen-Induced Arthritis Using a Dual Color ELISPOT Assay. J. Interf. Cytokine Res..

[21] Chokkalingam V., Tel J., Wimmers F., Liu X., Semenov S., Thiele J., Figdorb C., Huck W. (2013). Probing cellular heterogeneity in cytokine-secreting immune cells using droplet-based microfluidics. Lab Chip.

[22] Briones J., Okui Y., Espulgar W., Park J., Itotagawa E., Koyama S., Tamiya E., Takamatsu H., Saito M. (2023). The combination of hexagonal microfluidic devices and cell-based reporter cells allows detection of cytokine-producing cells at the single-cell level. Sens. Actuators B Chem..

[23] George J., Wang J. (2016). Assay of genome-wide transcriptome and secreted proteins on the same single immune cells by microfluidics and RNA sequencing. Anal. Chem..

[24] Choi J.R., Lee J.H., Xu A., Matthews K., Xie S., Duffy S.P., Ma H. (2020). Monolithic hydrogel nanowells-in-microwells enabling simultaneous single cell secretion and phenotype analysis. Lab Chip.

[25] Mariani S., Minunni M. (2014). Surface plasmon resonance applications in clinical analysis. Anal. Bioanal. Chem..

[26] Saleem I., Widger W., Chu W.-K. (2017). A new technique to detect antibody-antigen reaction (biological interactions) on a localized surface plasmon resonance (LSPR) based nano ripple gold chip. Appl. Surf. Sci..

[27] Laigre E., Goyard D., Tiertant C., Dejeu J., Renaudet O. (2018). The study of multivalent carbohydrate-protein interactions by bio-layer interferometry. Org. Biomol. Chem..

[28] Aoki H., Corn R.M., Matthews B. (2019). MicroRNA detection on microsensor arrays by SPR imaging measurements with enzymatic signal enhancement. Biosens. Bioelectron..

[29] dos Santos P.S.S., de Almeida J.M.M.M., Pastoriza-Santos I., Coelho L.C.C. (2021). Advances in Plasmonic Sensing at the NIR—A Review. Sensors.

[30] Huang T., Nallathamby P.D., Xu X.-H.N. (2008). Photostable Single-Molecule Nanoparticel Optical Biosensors for Real-Time Sensing of Single Cytokine Molecules and Their Binding Reactions. J. Am. Chem. Soc..

[31] Seplúveda B., Angelomé P.C., Lechuga L.M., Liz-Marzán L.M. (2009). LSPR-based nanobiosensors. Nano Today.

[32] Chen P., Chung M.T., McHugh W., Nidetz R., Li Y., Fu J., Cornell T.T., Shanley T.P., Kurabayashi K. (2015). Multiplex Serum Cytokine Immunoassay Using Nanoplasmonic Biosensor Microarrays. ACS Nano.

[33] Wang Y., Zhou J., Li J. (2017). Construction of Plasmonic Nano-Biosensor-Based Devices for Point-of-Care Testing. Small Methods.

[34] Taghavi A., Rahbarizadeh F., Abbasian S., Moshaii A. (2020). Label-Free LSPR Prostate-Specific Antigen Immune-Sensor Based on GLAD-Farbricated Silver Nano-columns. Plasmonics.

[35] Ali R.A.M., Mita D., Espulgar W., Saito M., Nishide M., Takamatsu H., Yoshikawa H., Tamiya E. (2020). Single Cell Analysis of Neutrophils NETs by Microscopic LSPR Imaging System. Micromachines.

[36] Zhu C., Luo X., Espulgar W.V., Koyama S., Kumanogoh A., Saito M., Takamatsu H., Tamiya E. (2020). Real-Time Monitoring and Detection of Single-Cell Level Cytokine Secretion Using LSPR Technology. Micromachines.

[37] He J., Brimmo A.T., Qasaimeh M.A., Chen P., Chen W. (2017). Recent Advances and Perspectives in Microfluidics-Based Single-Cell Biosensing Techniques. Small Methods.

[38] Wang C., Cai Y., MacLachlan A., Chen P. (2020). Novel Nanoplasmonic-Structure-Based Integrated Microfluidic Biosensors for Label-Free In Situ Immune Functional Analysis. IEEE Nanotechnol. Mag..

[39] Mazutis L., Glibert J., Ung W.L., Weitz D.A., Griffiths A.D., Heyman J.A. (2013). Single-cell analysis and sorting using droplet-based microfluidics. Nat. Protoc..

[40] Yin M., Marshall D. (2012). Microfluidics for single cell analysis. Curr. Opin. Biotechnol..

[41] Thompson A.M., Paguirigan A.L., Kreutz J.E., Radich J.P., Chiu D.T. (2014). Microfluidics for single-cell genetic analysis. Lab Chip.

[42] Briones J.C., Espulgar W.V., Koyama S., Yoshikawa H., Park J., Naito Y., Kumanogoh A., Tamiya E., Takamatsu H., Saito M. (2020). A Microfluidic Platform for Single Cell Fluorometric Granzyme B Profiling. Theranostics.

[43] Ide H., Espulgar W.V., Saito M., Aoshi T., Koyama S., Takamatsu H., Tamiya E. (2021). Profiling T cell interaction and activation through microfluidics-assisted serial encounter with APCs. Sens. Actuators B Chem..

[44] Ide H., Aoshi T., Saito M., Espulgar W.V., Briones J.C., Hosokawa M., Matsunaga H., Arikawa K., Takeyama H., Koyama S. (2023). Linking antigen specific T-cell dynamics in a microfluidic chip to single cell transcription patterns. Biochem. Biophys. Res. Commun..

[45] Wei S.-C., Hsu M.N., Chen C.-H. (2019). Plasmonic droplet screen for single-cell analysis. Biosens. Bioelectron..

[46] Funari R., Chu K.Y., Shen A.Q. (2020). Detection of antibodies against SARS-CoV-2 spike protein by gold nanospikes in an opto-microfluidic chip. Biosens. Bioelectron..

[47] Siao Y.-J., Peng C.-C., Tung Y.-C., Chen Y.-F. (2022). Comparison of Hydrogen Peroxide Secretion from Living Cells Cultured in Different Formats Using Hydrogel-Based LSPR Substrates. Front. Bioeng. Biotechnol..

[48] Saito M., Kitamura A., Murahashi M., Yamanaka K., Hoa L.Q., Yamaguchi Y., Tamiya E. (2012). Novel Gold-Capped Nanopillars Imprinted on a Polymer Film for Highly Sensitive Plasmonic Biosensing. Anal. Chem..

[49] Luo X., Zhu C., Saito M., Espulgar W.V., Dou X., Terada Y., Obara A., Uchiyama S., Tamiya E. (2020). Cauliflower-Like Nanostructured Localized Surface Plasmon Resonance Biosensor Chip for Cytokine Detection. Bull. Chem. Soc. Jpn..

[50] Terada Y., Obara A., Takamatsu H., Espulgar W.V., Saito M., Tamiya E. (2021). Au-capped Nanopillar Immobilized with a Length-Controlled Glycopolymer for Immune-Related Protein Detection. Appl. Bio Mater..

