# Supplementary material for: Development of Nano–Micro Fused LSPR Chip for In Situ Single-Cell Secretion Analysis
Source: Micromachines (Basel). 2023 Jul 11;14(7):1404. doi: 10.3390/mi14071404 (PMC10384685; doi:10.3390/mi14071404)
Supplement: Supplementary file 1 [file micromachines-14-01404-s001.zip › micromachines-2480152-supplementary.pdf]

micromachines

Supplementary Information: Development of Nano–Micro Fused LSPR Chip for In Situ Single-Cell Secretion Analysis

Yuhei Terada [1,2,3], Ain Obara [2,3], Jonathan Campos Briones [4], Xi Luo [2,3], Wilfred Villariza Espulgar [2,3], Masato Saito [4,*], Hyota Takamatsu [5,6] and Eiichi Tamiya [2,7]

[1] Environmental Management Research Institute (EMRI), Department of Energy and Environment, National Institute of Advanced Industrial Science and Technology (AIST), Tsukuba 305-8569, Ibaraki, Japan
[2] Advanced Photonics and Biosensing Open Innovation Laboratory, AIST-Osaka University, Suita 565-0871, Osaka, Japan
[3] Department of Applied Physics, Graduate School of Engineering, Osaka University, Suita 565-0871, Osaka, Japan
[4] Life and Medical Photonics Division, Institute for Open and Transdisciplinary Research Initiatives, Osaka University, Suita 565-0871, Osaka, Japan
[5] Department of Respiratory Medicine and Clinical Immunology, Graduate School of Medicine, Osaka University, Suita 565-0871, Osaka, Japan
[6] Laboratory of Autoimmune Diseases, Department of Clinical Research Center for Autoimmune Diseases, NHO Osaka Minami Medical Center, Kawachinagano 586-8521, Osaka, Japan
[7] SANKEN, Osaka University, Ibaraki 567-0047, Osaka, Japan
* Correspondence: saitomasato@ap.eng.osaka-u.ac.jp

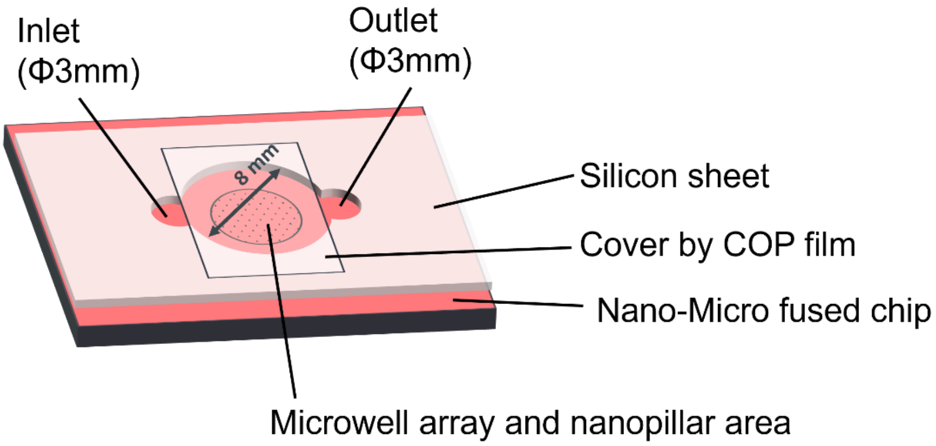

Figure S1. Schematic illustration of the of nano-micro fused chip set-up for cell trapping.
